# Late relapsing germ cell tumors with elevated tumor markers

**DOI:** 10.1007/s00345-021-03833-z

**Published:** 2021-09-13

**Authors:** Yue Che, Achim Lusch, Christian Winter, Robert Große Siemer, Carolin Buddensieck, Peter Albers, Andreas Hiester

**Affiliations:** 1 Department of Urology, Medical Faculty, Heinrich Heine University Düsseldorf, Moorenstr. 5, 40225 Düsseldorf, Germany; 2 Department of Urology, Helios University Hospital Wuppertal, Wuppertal, Germany

**Keywords:** Germ cell tumor, Late relapse, Surgery, Tumor marker

## Abstract

**Purpose:**

Late relapsing germ cell tumors (LR-GCT) are considered a rare distinct biologic entity as their clinical presentation and response to treatment is different to early recurrences. While serum tumor markers (AFP and ß-HCG) play an important role at the time of first diagnosis to correctly classify prognosis and treatment of germ cell tumors, they may not have the same significance in a late relapse situation.

**Patients and methods:**

Thirty-seven patients with LR-GCT with elevated serum tumor markers were identified in our database. Twenty-six patients underwent primary surgical resection of the late relapsing tumor. Eleven patients received salvage chemotherapy and a post-chemotherapy residual tumor resection. Serum tumor markers, histological findings and oncological outcome were analyzed.

**Results:**

In the histopathological specimen, viable cancer was found in 20 cases (54%) and teratoma was found in 16 cases (43%). In nine cases (24%), a somatic-type malignant transformation was present. In 19 of 37 patients (51.4%), the late relapse specimen presented a histological type of GCT, which was not present in the primary histology. Twenty-two patients (59.5%) were included in follow-up analysis. Mean and median follow-up time was 62.2 and 53 months, respectively. Seventeen patients (77.3%) suffered a relapse or had progressive disease after LR therapy. Five patients (22.7%) have been relapse-free after LR therapy (mean FU 61.6 months). Ten patients died of disease during follow-up (45.5%) and had a mean time from LR to death of 66.4 months. Eleven patients were alive at last follow-up (mean FU 62.2 months). Relapse and survival rate were similar between patients who received primary resection of LR tumor and patients who received salvage chemotherapy followed by surgery.

**Conclusion:**

Patients with a late relapsing germ cell tumor and elevated markers have a poor prognosis and a high risk for another relapse independent on primary treatment. The histological type and aggressiveness of a late relapsing tumor cannot be predicted with serum tumor marker levels at the time of diagnosis of LR. In up to 54% of cases, primary histology did not coincide with LR histology. Therefore, we propose primary surgical resection of a late relapsing tumor if a complete resection is feasible in order to gain exact histology and tailor further treatment.

## Introduction

In germ cell tumors (GCT), a tumor recurrence more than 2 years following initial treatment including chemotherapy is considered a late relapse (LR). It is a rare situation with a reported incidence of 3.2% in non-seminomatous GCT (NSGCT) and 1.4% in seminoma [1]. Late relapsing GCT (LR-GCT) are considered a distinct biologic entity, which is still not fully understood. Biologically, a LR-GCT is a tumor coming from an almost inactive or dormant state, which might have been triggered under cellular stress during cisplatin therapy. While resected specimens may contain all histological types of GCT [2], teratoma is the predominant one. This is related to the chemoresistant nature of teratomas.

Surgery has been proposed as the most important tool in the treatment of LR [1, 3, 4] as LR tumors are often chemorefractory [5]. Because of the heterogeneous biology of LR-GCT, the general management recommendation is to completely resect the LR tumor or obtain a representative biopsy before salvage therapy is carried out.

In GCT, serum tumor markers (STM) play an important role at the time of first diagnosis to correctly classify the tumor and yield information about the prognosis [6]. Treatment is adjusted to marker levels, and markers are necessary to monitor treatment response. AFP is secreted by 40–60% of patients with embryonal cell carcinoma and yolk sac tumor, while HCG is elevated in 10–20% of patients with choriocarcinoma [7]. At the time of recurrence, GCT are frequently treated with salvage chemotherapy when STM are elevated. Only patients with marker-negative recurrence have an undoubtable indication for primary surgery. In late relapsing patients with elevated markers, the question of primary surgery or primary chemotherapy remains unsolved.

In this retrospective study, we aimed to investigate the significance of tumor marker elevation at the time of diagnosis of a LR. In this case series we analyzed 37 patients who had a LR with elevated tumor markers and underwent primary surgical resection of the tumor or residual tumor resection after chemotherapy.

## Patients and methods

### Study population

After institutional review board, patients underwent chemotherapy or primary surgical resection of a late relapsing germ cell tumor with elevated STMs. LR was defined as a relapse 2 years or later after last chemotherapy. Elevated STMs were defined by our laboratory as a level of human chorionic gonadotropin (ß-HCG) equal to or greater than 2.0 mIU/ml and/or a level of alpha-fetoprotein (AFP) equal to or greater than 7.0 µg/l. Patient characteristics are shown in Table 1.

**Table 1:** Patients’ characteristics

All patients characteristic	*n* =	Primary LR section *n* =	Chemotherapy with PC-RTR *n* =
Primary histology			
Seminoma	0		
NSGCT	37	26	11
Ectragonadal			
Yes	5	4	1
Initial clinical stage			
IS	1		1
II	19	14	5
III	17	12	5
Initial IGCCG			
Good	16	11	5
Intermediate	5	3	2
Poor	8	6	2
Unknown	8	6	2
Site of late recurrence			
Retroperitoneal	36	25	11
Pulmonal	4	0	4
Mediastinum	5	4	1
Liver	2	0	2
Bone	2	0	2
Superaclavicular	1	1	0
Time from end of last treatment to relapse			
Mean	114 months	120 months	99 months
Median	96 months	101 months	96 months
Range	30–304 months	30–304 months	54–160 months
Markers before chemotherapy			
AFP positive, *n* =			10
AFP (μg/l), mean			20,328
AFP median			16,357
AFP, range			31–50,907
AFP, IQR			139–33,000
HCG positive, *n* =			1
HCG (mlU/ml)			180
Markers before surgery			
AFP positive, *n* =		25	9
AFP (μg/l), mean		492.25	1104
AFP median		38.45	138
AFP, range		7.4–5480	2.6–7000
AFP, IQR		12.8–135.25	47.75–945.5
HCG positive, *n* =		1	0
HCG (mlU/ml)		2.1	
Histology			
EC	9	6	3
Yolk sac	10	8	2
Choriocarcinoma	0	0	0
Teratoma	24	15	9
Seminoma	2	1	1
Somatic-type malignant transformation	9	5	4year
Follow-up after late relapse			
Incomplete follow-up data	14		
Relapse after late relapse	17		
Dead	10		
Survived (follow-up > 2 years)	11		
Follow-up time, mean	59 months		
Follow-up time, median	49 months		

*NSGCT* non-seminomatous germ cell tumor, *HCG* human chorionic gonadotropin, *AFP* alpha-fetoprotein

### Statistical methods

Means, medians and interquartile ranges were calculated for measured variables, respectively. Fisher’s exact test or *t* tests were performed as indicated in the results section. Statistical tests were performed using online tools provided by VassarStats. All tests were set with a significance level at *p* value < 0.05.

## Results

Between July 2008 and December 2020, 671 retroperitoneal lymph-node dissections (RPLND) were performed in our referral center. We queried our database for patients who had a LR-GCT with elevated STMs. Thirty-seven patients were identified. Twenty-six patients underwent primary surgical resection of the LR. The decision for a surgical approach was discussed in the interdisciplinary tumor board with the primary goal of a complete resection of all lesions. Eleven patients received second-line or salvage chemotherapy and a post-chemotherapy residual tumor resection.

### Previous therapy, relapse location and time to relapse

**Fig. 1:**
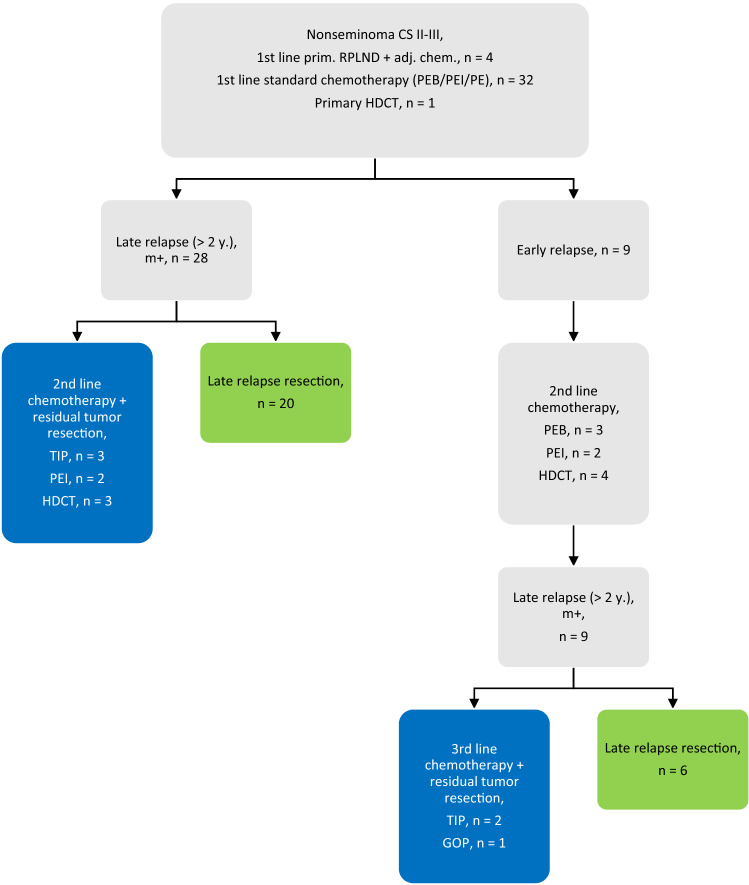
Previous therapies before late relapse and late relapse therapy. Blue box: salvage chemotherapy + PC-RTR, Green box: primary resection, PEB: cisplatin, etoposide, bleomycin, PEI: cisplatin, etoposide, ifosfamide, PE: cisplatin, etoposide, HDCT: high-dose chemotherapy, m+ : serum marker positive, y.: years, CS: clinical stage

All patients had a NSGCT with a clinical stadium II (IS) or III at first diagnosis. Four patients underwent primary RPLND with adjuvant chemotherapy with two cycles of bleomycin, etoposide and cisplatin (BEP). Thirty-two patients received standard first-line chemotherapy, and one patient received primary high-dose chemotherapy at diagnosis (Fig. 1). Twenty-eight patients had a LR with elevated STMs after first-line therapy. Of these 28 patients, eight patients received second-line chemotherapy with subsequent post-chemotherapy residual tumor resection (PC-RTR). Twenty patients underwent primary resection for LR tumor.

Nine patients had an early relapse after first-line therapy and received second-line therapy. These nine patients had a LR with elevated STMs after second-line therapy. Three patients received third-line chemotherapy and residual tumor resection. Six patients underwent primary LR resection.

The most frequent site of a LR was the retroperitoneum (36/37, 97%). Other relapse sites were the lung (4), the mediastinum (5), liver (2), bone (2) and supraclavicular (1) (Table 1).

Mean and median time from last therapy to LR was 114 and 96 months, respectively (med. IQR 62–127). The longest time to LR was 304 months (25 years).

### Serum tumor markers and histology (Table 2)

**Table 2:**
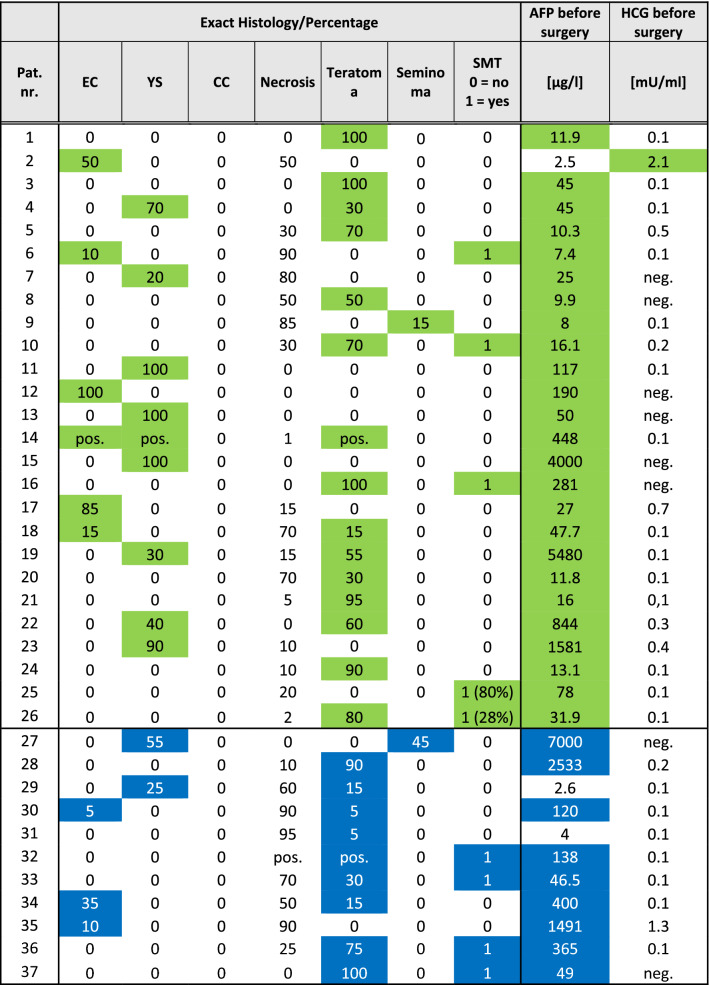
Histology and tumor markers before surgery

Primary LR resection group: green. LR chemotherapy with PC RTR group: blue. *EC* embryonal carcinoma, *YS* yolk sac, *CC* choriocarcinoma, *SMT* somatic-type malignant transformation

All patients had elevated STM at the time of LR. AFP was elevated in 35 patients, and ß-HCG was elevated in two patients. This unequal distribution can be explained by the main evidence of teratoma and yolk sac in the histological specimens.

Patients who received second-line or salvage chemotherapy had a mean AFP level of 20,328 µg/l (IQR 139–33,000 µg/l). Nine patients of this group still had elevated STMs before PC-RTR with a mean AFP level of 1104 µg/l (IQR 48–946 µg/l). Patients who received primary surgery had a mean AFP level of 493 µg/l (IQR 13–135 µg/l) at LR. AFP level was significantly higher in patients who received primary chemotherapy than in patients who underwent primary surgery (*p* = < 0.0001).

In the histopathological specimen, viable cancer was found in 20 cases (54%) and teratoma was found in 16 cases (43%). In 9 cases (24%), a somatic-type malignant transformation was present. The exact histological findings are shown in Table 2.

Looking closer at the preoperative STM levels, specimens containing yolk sac tumor had significantly higher AFP levels (mean AFP level = 1801 µg/l) than specimens containing embryonal carcinoma (mean AFP level 303 µg/l, one-tailed *t* test, *p* = 0.0412) or teratoma (mean AFP level 224 µg/l, one-tailed t test, *p* = 0.035).

In 19 of 37 patients (51%), the LR specimen presented a histological type of GCT, which was not present in the primary histology. In four patients, the primary histology was only known as NSGCT without any further specification (Table 3).

**Table 3:**
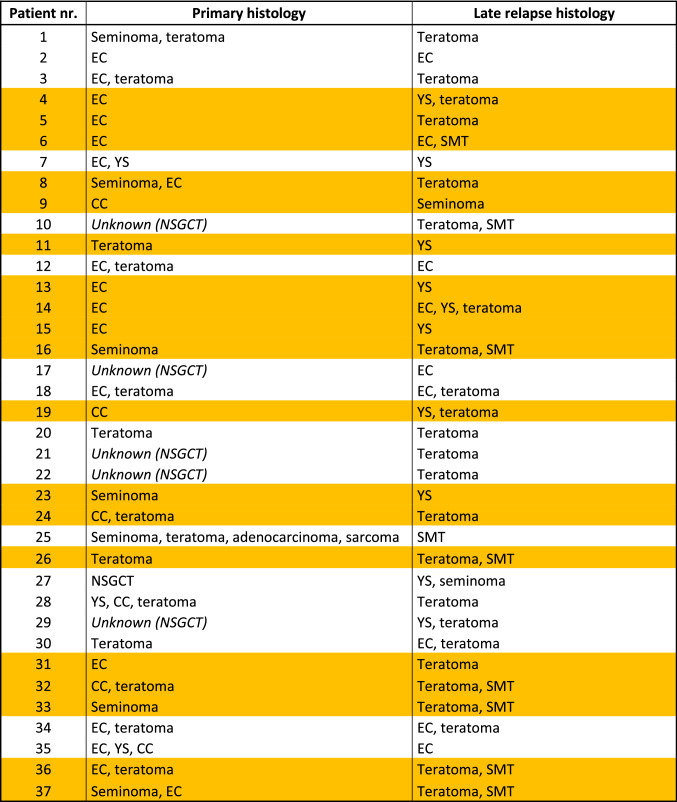
Comparison of primary and late relapse histology for each patient

In total, 54% of patients’ primary histology differs from late relapse histology (highlighted)

*NSGCT* non-seminomatous germ cell tumor, *EC* embryonal carcinoma, *YS* yolk sac, *CC* choriocarcinoma, *SMT* somatic-type malignant transformation

### Follow-up and outcome

Fifteen patients (41%) were lost to follow-up (FU) or had insufficient follow-up time (< 2 years) to be included in follow-up analysis. Twenty-two patients (60%) were included in follow-up analysis. Mean and median FU time was 62 and 53 months, respectively. Relapse and survival rate were similar in patients who received primary resection of LR tumor and patients who received salvage chemotherapy (Table 4). The differences between therapy groups were not statistically significant (Fisher test, *p* = 0.66 and 1, respectively). Seventeen patients (77%) suffered a relapse or had progressive disease after LR therapy, 80% after primary resection and 71% after salvage chemotherapy with subsequent PC-RTR. Five patients (23%) have been relapse-free after LR therapy (mean FU 62 months), three patients after primary resection and two after salvage chemotherapy. Ten patients died of disease during FU (45.5%) and had a mean time from LR to death of 66 months, six patients after primary resection (40%) and five after salvage chemotherapy (57%). Eleven patients were alive at last FU (mean FU 62.2 months), eight patients after primary resection (53%) and three after salvage chemotherapy (43%).

**Table 4:**
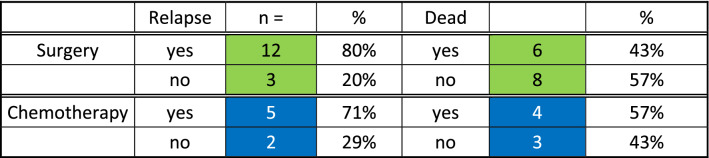
Outcome comparison of therapy groups

Patients who presented viable cancer (embryonal carcinoma, yolk sac and seminoma) or somatic-type malignant transformation had a relapse rate of 90% (9/10) and 80% (4/5), respectively, while patients who presented post-pubertal teratoma had a relapse rate of 57% (4/7). Survival rate of patients with viable cancer and somatic-type malignant transformation was 33% (6/9) and 60% (2/5), respectively. Teratoma patients had a more favorable survival rate at 71% (5/7).

There was no significant difference in preoperative AFP levels between patients who suffered a relapse (mean AFP 926 µg/l) and patients who were relapse-free (mean AFP 1181 µg/l) after LR therapy (two-tailed *t* test, *p* = 0.84). AFP levels did not differ significantly either for patients who have died of disease (mean AFP of patients who died 1437 µg/l, mean AFP for patients who are still alive 577 µg/l, two-tailed *t* test, *p* = 0.34).

## Discussion

Comparing patients with primary resection of LR-GCT to those who received salvage chemotherapy with subsequent PC-RTR showed no significant difference in relapse rate or survival (*p* = 0.66). The poor prognosis of late relapsing tumors has been stated in numerous previous reports [1, 3, 4].

Patients who suffered from disease progression or a relapse after LR therapy and died of disease had a prolonged course from LR until death (> 5 years). This suggests that LR-GCT with elevated STMs are in most cases slowly proliferating tumors and resistant to salvage therapy. The level of AFP-elevation was not predictive of outcome or histological type of a LR-GCT regardless of LR therapy. Except choriocarcinoma, all types of malignant GCT were present in our histological findings with a high incidence of somatic-type malignant transformation (24%). This proves the heterogeneous nature of late relapsing tumors.

Previous studies have found that patients with teratoma at the time of LR had a better outcome compared to patients presenting with viable cancer [2, 8, 9]. In our series, patients with teratoma had a 71% cancer-specific survival if somatic-type malignant transformation was not present. Patients who presented with viable cancer had a considerably lower survival rate at 33%.

Oldenburg et al. reported about the incidence of marker elevation in LR tumors: AFP was elevated in 49% and ß-HCG in 24% of the cases [1]. Thirty-five of 37 of our patients had an AFP elevation, while only two patients showed ß-HCG-elevation. The low number of ß-HCG-elevated LR tumors in our cohort is probably attributed to bias. In our database, we only record patients who underwent surgery either as primary treatment or as secondary resection after chemotherapy. Choriocarcinoma is generally a chemosensitive tumor and, thus, underrepresented in patients with LR [10].

A remarkable finding is that although teratoma is normally characterized by normal STM [11], we found AFP elevation in 16 teratoma patients. While seven teratoma patients also presented somatic-type malignancy components, two patients with somatic-type malignancy did not show teratoma components. Tu et al. reported that patients with yolk sac–seminoma in primary histology were predisposed to undergo somatic transformation [15]. We could not observe this association of primary histology with somatic transformation in LR (see Table 3). Although a low level of AFP-elevation can be due to non-neoplastic reasons, four teratoma patients showed very high AFP levels (135, 281, 365 and 2533 µg/l). As AFP has been used as a marker of endodermal stem cell differentiation [12], we hypothesize that AFP could have been induced during the endodermal differentiation of an embryonal carcinoma into the three germ layers (teratoma). Alternatively, the AFP expression might have originated from undetected yolk sac tumor cells. This hypothesis may be applied even more so to those presenting with somatic-type malignancy because sarcomatous somatic-type malignancy has been reported to derive from yolk sac tumor lineage [13, 14].

A major limitation of this study is that the indication for primary resection or chemotherapy was based on individual considerations. Some patients were referred to our center for post-chemotherapy surgery after undergoing chemotherapy in another center. Thus, patients with a priori resectable findings underwent surgery, while patients with non-resectable findings, such as bone or hepatic metastases, and therefore worse prognosis per se, received chemotherapy. There is also a clear difference in the median STM levels before primary LR resection or chemotherapy (Table 1, *t* test, two-tailed, *p* = < 0.0001). A second major limitation is the high percentage of patients who were lost to follow-up. This fact reduces the validity of our follow-up analysis.

Nevertheless, we could show that in up to 54% of the cases the primary histology did not coincide with the findings on LR histology. On the one hand, patients with teratoma in the LR specimen in particular benefit from surgical treatment, as chemotherapy is not effective for this type of germ cell tumor. In our series, even some pure teratomas and somatic-type malignancies showed highly elevated STMs before surgery (see Table 2). Therefore, our recommended strategy is to perform primary surgery regardless of STM level if lesions are resectable to exclude pure teratoma or somatic-type malignancies in order to prevent unnecessary chemotherapy. On the other hand, even patients with completely resected LR had an unexpectedly high recurrence and, consecutively, a low cancer-specific survival rate. This new information on the outcome of patients with completely resected LR and elevated markers should guide the shared decision making of treatment and ask for close follow-up with early systemic salvage treatment if possible.

## Conclusions

Patients with a late relapsing germ cell tumor and elevated markers have an unexpectedly poor prognosis and a high risk for another relapse, independent of treatment, even if completely resected with primary surgery. The histological type and aggressiveness of a late relapsing tumor cannot be predicted with serum tumor marker levels at the time of diagnosis of LR. In up to 54% of cases, primary histology did not coincide with LR histology. Therefore, we propose primary surgical resection of a late relapsing tumor with elevated markers if a complete resection is feasible in order to prevent unnecessary chemotherapy in patients with teratoma and somatic-type malignancies. Since every second patient even with complete resection and marker normalization experiences relapse and faces a poor survival, novel systemic treatment options need to be developed.

## Declarations

### Disclaimer

We confirm that the manuscript has been read and approved by all named authors and that there are no other persons who satisfied the criteria for authorship but are not listed.
